# Retinal vasculitis associated with pyoderma gangrenosum: a case report

**DOI:** 10.1007/s12348-012-0083-9

**Published:** 2012-06-06

**Authors:** Nilay Yuksel, Sengul Ozdek

**Affiliations:** 1 Department of Ophthalmology, Kahramanmaras State Hospital, Yörükselim Mah. Gazi Mustafa Kuscu Cad., Merkez, 46100 Kahramanmaras, Turkey; 2 Department of Ophthalmology, Gazi University Medical School, Ankara, Turkey

## Introduction

Pyoderma gangrenosum (PG) is an uncommon, idiopathic ulcerative neutrophilic inflammatory skin disease characterized by variable clinical presentation, and the cause of this disease is uncertain [1]. Diagnosis of pyoderma gangrenosum is clinical, and histopathology is not specific and depends on exclusion of other causes of cutaneous ulceration [2]. Clinically it starts with sterile pustules that rapidly progress and become painful ulcers of different size with violaceous borders. The legs are most commonly affected, but other parts of the skin and mucous membranes may also be involved [3].

PG commonly occurs between 25 and 54 years of age and occurs rarely in children. The estimated incidence of PG is approximately 3–10 patients per 1,000,000 population per year [4].

Approximately 50 % of patients have an associated systemic disease, commonly inflammatory bowel disease (IBD), ulcerative colitis, arthritis, hematological and lymphoreticular malignancies, while in another 40–50 % patients, no underlying disease is found. The disease is recurrent in approximately 30 % of patients [5, 6]. The management of PG is treatment of underlying systemic medical illness and judicious use of immunosuppressants.

## Case report

**Fig. 1:**
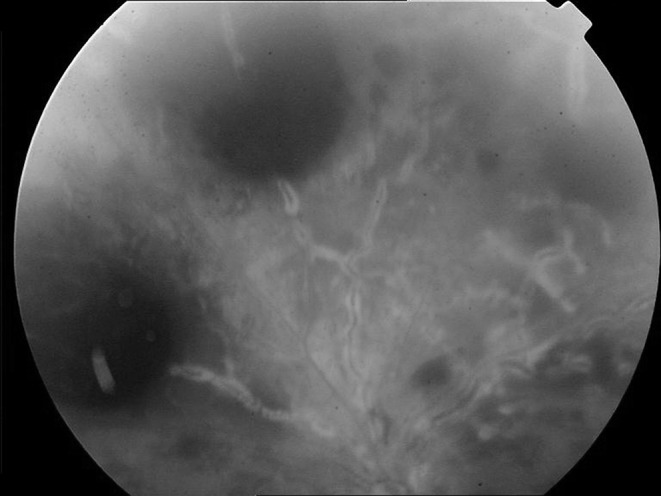
Fundus photograph of the left eye at presentation. Left eye showed large preretinal, intraretinal and subretinal hemorrhages involving the inferior part of the macula, inferior temporal arcade, nasal and superior parts of retina and edema at the optic nerve head. Vascular changes included significantly increased tortuosity and diffuse perivascular sheathing

**Fig. 2:**
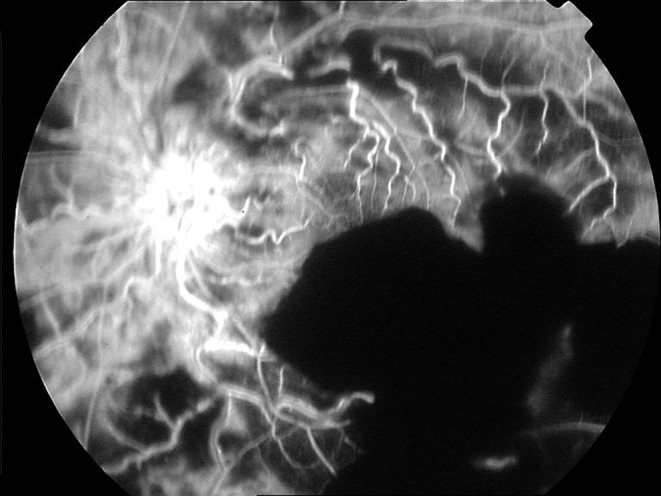
FFA of left eye revealed some capillary non-perfusion areas and blocked fluorescence in hemorrhagic areas

A 24-year-old male patient presented with a sudden visual loss which started a few hours earlier in his left eye. His medical history revealed PG for the last 3 years. He was on treatment with cyclosporin A (2 × 150 mg) and deflazacort (1 × 30 mg) for maintenance therapy of PG. In his ophthalmologic examination, visual acuity was 20/25 OD and 20/1200 OS. Slit lamp examination was unremarkable except bilateral mild posterior subcapsular cataracts. Intraocular pressures, pupil examination and extraocular muscle movements were normal in both eyes. Fundus examination of the right eye showed healthy retina, vitreous, optic nerve head and retinal vasculature, and left eye showed large preretinal, intraretinal and subretinal hemorrhages involving the inferior part of the macula, inferior temporal arcade, nasal and superior parts of retina and edema at the optic nerve head. Vascular changes included significantly increased tortuosity and diffuse perivascular sheathing (Fig. 1). There was no vitritis. Fundus fluorescein angiogram (FFA) of left eye revealed delayed filling of peripheral vessels, late staining of vessels and optic nerve head together with some capillary non-perfusion areas and blocked fluorescence in hemorrhagic areas (Fig. 2). FFA of right eye was normal.

**Fig. 3:**
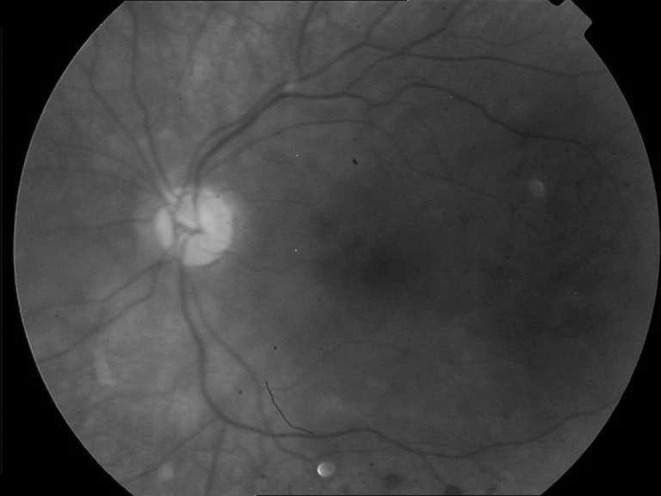
At the 18th month visit, the left fundus was almost normal except from some vitreous opacity inferiorly without any vascular finding

The patient was evaluated for tuberculosis with PPD and chest X-ray, syphilis with VDRL, sarcoidosis with chest X-ray and serum and urine calcium levels, Behcet’s disease with pathergy test, spondyloarthropathies with HLA-B27, SLE and other collagen vascular disorders, IBD and prothrombotic conditions. All of the tests and examinations were negative for these diseases. The patient was assessed as retinal vasculitis due to PG. The dose of deflazacort which is a steroid was increased to 60 mg by his dermatologist after the tests. The hemorrhages started to decrease at the third week visit and totally resolved within 6 months. Steroid dose was decreased to a maintenance dose of 10 mg/day. Visual acuity in left eye increased to 20/25 at the eighth month and started to decrease thereafter because of the posterior subcapsular cataracts to become 20/50 at the last visit. At the 18th month follow-up, fundus evaluation revealed only some vitreous opacity inferiorly without any vascular finding in the left eye (Fig. 3).

## Discussion

Pyoderma gangrenosum is an uncommon chronic ulcerative condition, the etiology of which is poorly understood [7]. Common disease associations are inflammatory bowel disease, arthritis, monoclonal gammopathy, leukemia, immune deficiency state and HIV infection [5, 6].

Systemic steroids and cyclosporine A either alone or in combination are considered to be the first line of treatment [8]. Other agents used include dapsone, clofazimine, thalidomide, azathioprine and mycophenolate mofetil [9].

Ophthalmic findings associated with PG are rare [7]. PG-associated nodular scleritis [10], orbital inflammation [10], eyelid involvements [11–13] and peripheral ulcerative keratitis [14] have been reported. In the vast majority of cases, immunosuppressive therapy had been successful.

Despite immunosuppressive therapy with prednisolone and cyclosporine A, a case with destruction of the orbital contents with subsequent perforation of the eye went evisceration [7]. Evisceration was performed together with hyperbaric oxygen therapy pre- and postoperatively. Hyperbaric oxygen therapy helps to reverse the impaired neutrophil function and promotes fibroblasts to produce collagen [7].

Our patient's history consisted of chest, face and lower extremity papules that eventually ulcerated for 3 years, diagnosed PG based on the clinical aspects of the skin lesions, after exclusion of other specific ulcerative processes by his dermatologists. Histopathology of the lesions was unspecific (chronic ulcer), and he had no systemic disease associated. After ocular involvement occurred, detailed investigation was made for differential diagnosis of retinal vasculitis. Clinical and laboratory tests were negative for other systemic diseases; thereafter, diagnosis was assessed as retinal vasculitis due to PG.

The only PG case associated with retinal vasculitis in the literature was a 49-year-old man who had some other bilateral ocular findings like limbal neovascularization, corneal thinning, extensive gutter formation at the corneal/scleral margin and corneal ulcerations [15]. Immunosuppressive therapy with prednisolone 10 mg daily, dapsone 100 mg daily, minocycline 100 mg daily and topical betamethasone did well in this particular case.

## Conclusion

In conclusion, in the absence of other common causes of retinal vasculitis, we presume that in this case the retinal vasculitis is being caused by PG which responds well to systemic steroid therapy. Early recognition of the manifestations can lead to the institution of appropriate therapy, thereby improving the patient's prognosis.
